# Diet–gut microbiota interaction Index and heart failure risk in diabetes and prediabetes: evidence from NHANES 2007–2018

**DOI:** 10.1093/eschf/xvag125

**Published:** 2026-05-04

**Authors:** Yuqing Huang, Yuling Yang, Jia Feng, Qiming Gong, Yueli Pu, Xia Fang, Yong Xu

**Affiliations:** Department of Endocrinology and Metabolism, The Affiliated Hospital of Southwest Medical University, No. 25 Taiping Street, Luzhou, Sichuan 646000, China; Metabolic Vascular Diseases Key Laboratory of Sichuan Province, Section 3, Zhongshan Road, Luzhou, Sichuan 646000, China; Department of Endocrinology and Metabolism, The Affiliated Hospital of Southwest Medical University, No. 25 Taiping Street, Luzhou, Sichuan 646000, China; Metabolic Vascular Diseases Key Laboratory of Sichuan Province, Section 3, Zhongshan Road, Luzhou, Sichuan 646000, China; Department of Endocrinology and Metabolism, The Affiliated Hospital of Southwest Medical University, No. 25 Taiping Street, Luzhou, Sichuan 646000, China; Metabolic Vascular Diseases Key Laboratory of Sichuan Province, Section 3, Zhongshan Road, Luzhou, Sichuan 646000, China; Department of Endocrinology and Metabolism, The Affiliated Hospital of Southwest Medical University, No. 25 Taiping Street, Luzhou, Sichuan 646000, China; Metabolic Vascular Diseases Key Laboratory of Sichuan Province, Section 3, Zhongshan Road, Luzhou, Sichuan 646000, China; Department of Endocrinology and Metabolism, The Affiliated Hospital of Southwest Medical University, No. 25 Taiping Street, Luzhou, Sichuan 646000, China; Metabolic Vascular Diseases Key Laboratory of Sichuan Province, Section 3, Zhongshan Road, Luzhou, Sichuan 646000, China; Department of Endocrinology and Metabolism, The Affiliated Hospital of Southwest Medical University, No. 25 Taiping Street, Luzhou, Sichuan 646000, China; Metabolic Vascular Diseases Key Laboratory of Sichuan Province, Section 3, Zhongshan Road, Luzhou, Sichuan 646000, China; Department of Endocrinology and Metabolism, The Affiliated Hospital of Southwest Medical University, No. 25 Taiping Street, Luzhou, Sichuan 646000, China; Metabolic Vascular Diseases Key Laboratory of Sichuan Province, Section 3, Zhongshan Road, Luzhou, Sichuan 646000, China

**Keywords:** DI-GM, Heart failure, Diabetes, Prediabetes, Gut–Heart Axis, NHANES

## Abstract

**Background and Aims:**

Diabetes and prediabetes markedly increase the risk of heart failure (HF), but the role of diet–gut microbiota interactions remains unclear. This study examined the association between the Dietary Index for Gut Microbiota (DI-GM) and HF risk among individuals with diabetes or prediabetes.

**Methods:**

Data were obtained from 15 219 adults with diabetes or prediabetes in the US National Health and Nutrition Examination Survey (NHANES) 2007–2018. DI-GM scores were calculated from two 24-h dietary recalls covering 14 food groups linked to gut microbiota. Associations between DI-GM and prevalent HF were estimated using weighted logistic regression and restricted cubic spline models, adjusting for demographic, lifestyle, and metabolic factors.

**Results:**

Participants had a mean age of 56.50 ± 15.68 years, and 48.14% were women. Higher DI-GM scores were independently associated with lower HF risk (adjusted odds ratio [OR] per 1-point increase = 0.93, 95% confidence interval [CI] 0.89–0.98; *P* = .005). Compared with scores 0–3, DI-GM ≥ 6 was linked to 27% lower HF risk (OR = 0.73, 95% CI 0.58–0.92; *P* = .007). The inverse association was stronger in prediabetes (OR = 0.89, 95% CI 0.82–0.96; *P* = .004) but not significant in diabetes.

**Conclusions:**

Higher DI-GM was associated with lower HF risk, particularly in prediabetes. Microbiota-related dietary patterns may play a role in HF prevention among metabolically at-risk populations.

## Graphical Abstract

**Graphical Abstract:**
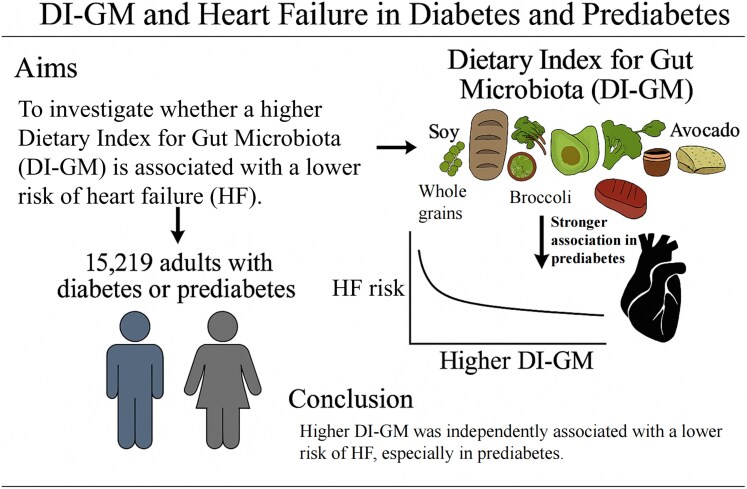

## Introduction

Diabetes mellitus (DM) remains a major global public health issue, with its prevalence continuing to rise. According to estimates from the International Diabetes Federation (IDF), approximately 537 million adults (aged 20–79 years), or 10.5% of the global adult population, are currently living with diabetes, and this number is projected to increase to 783 million by 2045.^1^ Prediabetes primarily includes impaired glucose tolerance (IGT; 2-hour plasma glucose 7.8–11.0 mmol/L [140–199 mg/dL]) and impaired fasting glucose (IFG; fasting plasma glucose 6.1–6.9 mmol/L [110–125 mg/dL]).^1^ Substantial epidemiological evidence has demonstrated that both diabetes and prediabetes are closely associated with the development of heart failure (HF). Recent studies have reported that individuals with prediabetes, even in the absence of clinically overt HF, may already exhibit subclinical myocardial dysfunction, such as reduced global longitudinal strain (GLS), suggesting the presence of early cardiac structural and functional impairment.^2^ Patients with diabetes have a 2–4-fold higher risk of developing HF compared with individuals without diabetes, while the risk is also significantly increased among those with prediabetes.^3^ The incidence of HF is particularly pronounced in the elderly, rising markedly after the age of 65, with a 5-year mortality rate reaching as high as 50%. This condition not only severely compromises quality of life but also imposes a substantial medical and societal burden.^4^ Therefore, early screening and preventive interventions for HF among individuals with diabetes and prediabetes are of critical importance.

Dietary habits play a pivotal role in the management of diabetes and prediabetes. Adherence to a healthy dietary pattern helps regulate blood glucose levels, alleviate cardiac load, and consequently reduce both the risk and severity of HF.^5^ Diet also influences the onset and progression of diabetes and its complications by modulating the gut microbiota. Conversely, gut microbiota dysbiosis is closely linked to insulin resistance, chronic inflammation, and impaired glucose metabolism.^6–8^

The dietary index for gut microbiota (DI-GM) is a novel dietary assessment tool designed to quantify the overall impact of diet on the gut microbiome. Derived from a systematic review of 106 studies, the DI-GM incorporates 14 food groups known to be associated with gut microbiota composition, including beneficial components (e.g. fermented dairy products, whole grains, dietary fibre) and detrimental components (e.g. red meat, high-fat diet). The score ranges from 0 to 13, reflecting the potential influence of diet on the gut microbial ecosystem. Unlike approaches focusing on individual nutrients or single food categories, DI-GM integrates multiple microbiota-related food groups to quantify diet–microbiota interactions, thereby facilitating the assessment of associations between overall dietary patterns and cardiovascular risk.

Although prior studies have explored the associations of dietary patterns or gut microbiota with HF, most evidence remains confined to single-factor analyses. Integrated approaches that quantitatively capture both dietary structure and microbiota-related effects are still scarce, especially in populations with impaired glucose metabolism. Moreover, there is a lack of simple and clinically applicable tools that reflect both dietary patterns and their potential microbiota-mediated impacts, limiting risk stratification for HF in individuals with diabetes or prediabetes.

However, evidence regarding the association between DI-GM and the risk of HF among individuals with diabetes and prediabetes remains scarce. Based on data from the National Health and Nutrition Examination Survey (NHANES, 2007–2018), this study aims to investigate the relationship between DI-GM and HF risk in patients with diabetes and prediabetes.

## Methods

### Study population and design

**Figure 1:**
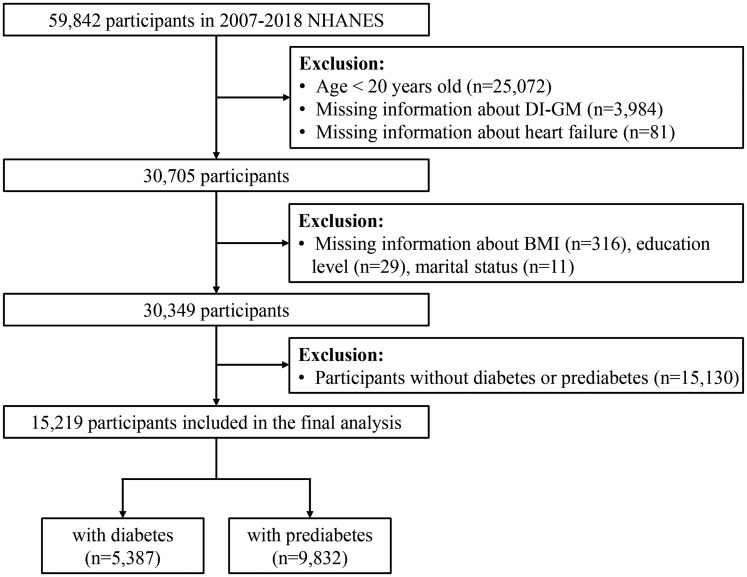
Flowchart of study participant selection

This study was based on data from the National Health and Nutrition Examination Survey (NHANES), a programme conducted by the Centers for Disease Control and Prevention (CDC) and the National Center for Health Statistics (NCHS). The study adhered to the Strengthening the Reporting of Observational Studies in Epidemiology (STROBE) guidelines, and all participants provided written informed consent approved by the NCHS Research Ethics Review Board. We extracted and combined data from six NHANES cycles (2007–2018) (https://www.cdc.gov/nchs/nhanes/), which included demographic characteristics, physical examination findings, and questionnaire information. To ensure the completeness and reliability of the results, we excluded participants younger than 20 years (*n* = 25 072), those without diabetes or prediabetes (*n* = 15 130), and those with missing data on DI-GM, HF, body mass index (BMI), education level, or marital status (*n* = 4421). A total of 15 219 participants were ultimately included in the analysis. The final study population had a mean age of 56.50 ± 15.68 years, and 48.14% were female. Among them, 35.40% had diabetes and 64.60% had prediabetes. Detailed baseline characteristics are presented in *Table 1*. The detailed participant selection process is illustrated in *Figure 1*. According to the American Diabetes Association (ADA) guidelines,^9^ diabetes was defined as meeting at least one of the following criteria: (1) self-reported diabetes diagnosis; (2) current use of insulin or oral hypoglycaemic agents; (3) glycated haemoglobin (HbA1c) ≥ 6.5%; (4) fasting blood glucose (FBG) ≥ 7.0 mmol/L; or (5) 2-hour plasma glucose (2hPG) ≥ 11.1 mmol/L following an oral glucose tolerance test (OGTT). Prediabetes was defined as meeting any of the following: (1) HbA1c 5.7–6.4%; (2) FBG 5.6–6.9 mmol/L; or (3) 2hPG 7.8–11.0 mmol/L. HF was defined based on self-reported physician diagnosis from the NHANES Medical Conditions Questionnaire (MCQ160B). Participants who answered ‘yes’ to the question, ‘Has a doctor or other health professional ever told you that you had congestive heart failure?’ were classified as having HF.

**Table 1:** Baseline characteristics of participants from NHANES 2007–2018

Characteristic	Overall (*n* = 15 219)	No-heart failure (*n* = 14 447)	Heart failure (*n* = 772)	*P*-value
Age (year)	56.50 ± 15.68	55.92 ± 15.67	67.51 ± 11.24	**<**.**001**
Waist circumference (cm)	104.36 ± 15.80	104.06 ± 15.67	110.11 ± 17.09	**<**.**001**
TC (mmol/L)	5.02 ± 1.12	5.05 ± 1.11	4.52 ± 1.14	**<**.**001**
HDL-C (mmol/L)	1.31 ± 0.39	1.31 ± 0.39	1.24 ± 0.39	**<**.**001**
HbA1c (%)	6.21 ± 1.46	6.20 ± 1.44	6.45 ± 1.84	**<**.**001**
DI-GM	4.58 ± 1.62	4.59 ± 1.62	4.38 ± 1.62	**<**.**001**
Beneficial to gut microbiota	1.96 ± 1.33	1.97 ± 1.33	1.70 ± 1.31	**<**.**001**
Unfavorable to gut microbiota	2.62 ± 1.07	2.62 ± 1.07	2.68 ± 1.12	.162
**Gender, *n* (%)**				.**013**
Male	7892 (51.86)	7458 (94.50)	434 (5.50)	
Female	7327 (48.14)	6989 (95.39)	338 (4.61)	
**Age strata, *n* (%)**				**<**.**001**
<40	2541 (16.70)	2524 (99.33)	17 (0.67)	
40–60	5385 (35.38)	5241 (97.33)	144 (2.67)	
≥60	7293 (47.92)	6682 (91.62)	611 (8.38)	
**Race, *n* (%)**				**<**.**001**
Mexican American	2449 (16.09)	2380 (97.18)	69 (2.82)	
Other Hispanic	1638 (10.76)	1570 (95.85)	68 (4.15)	
Non-Hispanic White	5836 (38.35)	5449 (93.37)	387 (6.63)	
Non-Hispanic Black	3632 (23.86)	3431 (94.47)	201 (5.53)	
Other Race	1664 (10.93)	1617 (97.18)	47 (2.82)	
**Education level, *n* (%)**				**<**.**001**
Less than high school	4375 (28.75)	4105 (93.83)	270 (6.17)	
High school or equivalent	3607 (23.70)	3408 (94.48)	199 (5.52)	
College graduate or above	7237 (47.55)	6934 (95.81)	303 (4.19)	
**Marital status, *n* (%)**				**<**.**001**
Married/Living with a partner	9228 (60.63)	8841 (95.81)	387 (4.19)	
Divorced/Separated/Widowed	4138 (27.19)	3811 (92.10)	327 (7.90)	
Never married	1853 (12.18)	1795 (96.87)	58 (3.13)	
**BMI, *n* (%)**				**<**.**001**
<25 kg/m^2^	2900 (19.06)	2786 (96.07)	114 (3.93)	
25–30 kg/m2	4967 (32.64)	4772 (96.07)	195 (3.93)	
≥30 kg/m2	7352 (48.31)	6889 (93.70)	463 (6.30)	
**PIR, *n* (%)**				**<**.**001**
<1.3	4481 (29.44)	4181 (93.31)	300 (6.69)	
1.3–3.5	6873 (45.16)	6512 (94.75)	361 (5.25)	
≥3.5	3865 (25.40)	3754 (97.13)	111 (2.87)	
**Alcohol use, *n* (%)**				.168
Yes	8193 (53.83)	7796 (95.15)	397 (4.85)	
No	7026 (46.17)	6651 (94.66)	375 (5.34)	
**Smoking, *n* (%)**				**<**.**001**
Never	7904 (51.94)	7598 (96.13)	306 (3.87)	
Previously	4390 (28.85)	4068 (92.67)	322 (7.33)	
Now	2925 (19.22)	2781 (95.08)	144 (4.92)	
**Hypertension, *n* (%)**				**<**.**001**
Yes	8712 (57.24)	8040 (92.29)	672 (7.71)	
No	6507 (42.76)	6407 (98.46)	100 (1.54)	
**Diabetes, *n* (%)**				**<**.**001**
Yes	5387 (35.40)	4911 (91.16)	476 (8.84)	
Pre	9832 (64.60)	9536 (96.99)	296 (3.01)	
**Glucose-lowering medication, *n* (%)**				
Insulin use	996 (6.54)	837 (84.04)	159 (15.96)	**<**.**001**
Oral medications	2728 (17.92)	2496 (91.50)	232 (8.50)	.232
**CHD, *n* (%)**				**<**.**001**
Yes	955 (6.28)	641 (67.12)	314 (32.88)	
No	14 264 (93.72)	13 806 (96.79)	458 (3.21)	
**Physical activity, *n* (%)**				**<**.**001**
Yes	5331 (35.03)	5117 (95.99)	214 (4.01)	
No	9888 (64.97)	9330 (94.36)	558 (5.64)	
**DI-GM, *n* (%)**				.**003**
0–3 score	3782 (24.85)	3569 (94.37)	213 (5.63)	
4 score	3771 (24.78)	3576 (94.83)	195 (5.17)	
5 score	3460 (22.73)	3266 (94.39)	194 (5.61)	
≥6 score	4206 (27.64)	4036 (95.96)	170 (4.04)	

Continuous variables: values are expressed as mean ± standard deviation.

Categorical variables: values are expressed as numbers (percentage).

TC, total cholesterol; HDL-C, high-density lipoprotein cholesterol; HbA1c, haemoglobin A1c; DI-GM, dietary index for gut microbiota; BMI, body mass index; PIR, poverty income ratio; CHD, coronary heart disease.

### Assessment of the dietary Index for gut Microbiota

DI-GM was calculated based on two 24-hour dietary recalls. The first recall was conducted at the Mobile Examination Center, and the second was collected through a telephone interview. This index reflects participants’ short-term dietary intake during the survey period. Given the cross-sectional design of NHANES, both the assessment of DI-GM and the ascertainment of HF status were conducted within the same survey cycle.

The DI-GM score incorporates 14 specific food groups or nutrients associated with gut health. Beneficial components include fermented dairy products, chickpeas, soy, whole grains, dietary fibre, cranberries, avocados, broccoli, coffee, and green tea, while red meat, processed meat, refined grains, and high-fat diets (defined as >40% of total energy intake from fat) were considered unfavourable components. Scoring was determined using sex-specific median intake levels. For each food or nutrient, a score of 1 was assigned if the participant's intake met the criterion, and 0 otherwise. The total DI-GM score was obtained by summing across all components, ranging from 0 to 13.^10^

### Covariates

Covariates were selected based on previous studies and included demographic characteristics: sex (male, female), age, race/ethnicity (Mexican American, non-Hispanic White, non-Hispanic Black, other), educational attainment (<high school, high school or equivalent, ≥college), marital status (married/living with partner, widowed/divorced/separated, never married), and poverty income ratio (PIR: <1.3, 1.3–3.5, >3.5). Anthropometric and metabolic factors included BMI (<25, 25–30, >30), waist circumference, smoking status (never, former, current), alcohol consumption (yes/no), total cholesterol (TC), high-density lipoprotein cholesterol (HDL-C), glycated haemoglobin (HbA1c), and physical activity level. In addition, history of diabetes, hypertension (systolic blood pressure ≥140 mmHg or diastolic blood pressure ≥90 mmHg), coronary heart disease (CHD), and glucose-lowering medication use (including insulin and oral hypoglycaemic agents) were also considered.

### Statistical analysis

To address missing covariate data, multiple imputation by chained equations (MICE) was applied. Participants were categorized into two groups according to the presence or absence of HF. Continuous variables were compared using weighted t-tests and expressed as mean ± standard deviation (SD), while categorical variables were compared using weighted chi-square tests and expressed as percentages.

To examine the association between DI-GM and HF, we constructed three weighted multivariable logistic regression models: Model 1 was unadjusted; Model 2 was adjusted for age, sex, and race/ethnicity; and Model 3 was further adjusted for all potential confounders. Models were constructed by adding covariates stepwise, and the association between DI-GM and HF was examined under different levels of adjustment to assess how the estimates changed. Stratified analyses were conducted separately for participants with diabetes and prediabetes, and restricted cubic splines (RCS) were applied to explore potential nonlinear relationships. The use of glucose-lowering medications (including insulin and oral agents) was examined across different DI-GM categories. In addition, medication use was further adjusted for on the basis of Model 3 in sensitivity analyses to evaluate the potential impact of residual confounding.

In addition, subgroup analyses and interaction tests were performed to evaluate the robustness and heterogeneity of the associations. Least absolute shrinkage and selection operator (LASSO) regression was used to identify key predictors for model construction, and model performance was comprehensively assessed using receiver operating characteristic (ROC) curves, decision curve analysis (DCA), and calibration plots.

All statistical analyses were performed using R software (version 4.3.3). A two-sided *P* value ≤ .05 was considered statistically significant.

## Results

### Characteristics of study participants

A total of 15 219 participants with diabetes or prediabetes were included in this study, comprising 7892 men (51.9%) and 7327 women (48.1%). Participants were divided into two groups based on the presence of HF: non-HF group (*n* = 14 447) and HF group (*n* = 772) (*Table 1*).

Significant differences were observed between the two groups across most variables, including age, waist circumference, lipid levels, glycaemic markers, and dietary index scores (all *P* < .05). Specifically, participants in the HF group were older, had larger waist circumferences, and exhibited higher HbA1c levels, whereas total cholesterol (TC) and high-density lipoprotein cholesterol (HDL-C) levels were lower. In addition, DI-GM scores were significantly lower in the HF group, suggesting a potential association between dietary patterns and HF risk among individuals with diabetes or prediabetes.

With respect to demographic and socioeconomic characteristics, the HF group had higher prevalence of hypertension, diabetes, and coronary heart disease, lower educational attainment, and a lower proportion of regular physical activity (*Table 1*). Notably, there were no statistically significant differences between the groups in alcohol consumption (*P* = .168) or scores for unfavourable dietary components (*P* = .162).

### Association between DI-GM and risk of heart failure among individuals with diabetes and prediabetes

As shown in *Table 2*, weighted logistic regression analyses revealed a significant inverse association between DI-GM and the risk of HF. In the unadjusted Model 1, each 1-point increase in DI-GM was associated with an 8% lower risk of HF (OR = 0.92, 95% CI: 0.88–0.96, *P* < .001). This association remained significant after full adjustment for sociodemographic, lifestyle, and metabolic factors in Model 3, with each 1-point increase in DI-GM linked to a 7% lower risk of HF (OR = 0.93, 95% CI: 0.89–0.98, *P* = .005). Subgroup analyses further demonstrated that compared with participants in the lowest DI-GM category (0–3 points), those with DI-GM ≥6 had a significantly reduced risk of HF (OR = 0.73, 95% CI: 0.58–0.92, *P* =.007), with a significant trend across categories (*P* for trend = .019).

**Table 2:** Relationship between DI-GM and odds of heart failure in diabetic and prediabetic participants

Characteristic		Model 1^a^		Model 2^b^		Model 3^c^	
		OR (95% CI)	*P*-value	OR (95% CI)	*P*-value	OR (95% CI)	*P*-value
**Diabetes and prediabetes participants**							
**Continuous DI-GM**		0.92 (0.88, 0.96)	**<**.**001**	0.89 (0.85, 0.93)	**<**.**001**	0.93 (0.89, 0.98)	.**005**
**DI-GM**	1 (0–3 score)	Ref		Ref		Ref	
	2 (4 score)	0.91 (0.75, 1.12)	.376	0.92 (0.75, 1.12)	.402	0.97 (0.78, 1.21)	.800
	3 (5 score)	1.00 (0.81, 1.22)	.963	0.96 (0.78, 1.18)	.719	1.04 (0.83, 1.30)	.727
	4 (≥6 score)	0.71 (0.57, 0.87)	**<.001**	0.61 (0.49, 0.75)	**<.001**	0.73 (0.58, 0.92)	.**007**
	*P* for trend	0.91 (0.85, 0.97)	**.004**	0.87 (0.81, 0.93)	**<.001**	0.92 (0.86, 0.99)	.**019**
Beneficial to gut microbiota		0.85 (0.81, 0.90)	**<.001**	0.85 (0.80, 0.90)	**<.001**	0.88 (0.83, 0.94)	**<.001**
Unfavorable to gut microbiota		1.05 (0.98, 1.12)	.162	0.98 (0.91, 1.04)	.472	1.02 (0.94, 1.09)	.678

DI-GM, dietary index for gut microbiota; PIR, poverty income ratio; BMI: body mass index; TC, total cholesterol; CHD: coronary heart disease; OR: odds ratio; 95% CI: 95% confidence interval.

^a^Model 1: no covariates were adjusted.

^b^Model 2: adjusted for gender, age, and race.

^c^Model 3: adjusted for gender, age, race, education level, marital status, PIR, BMI, waist circumference, smoking status, alcohol consumption, TC, diabetes, hypertension, CHD, physical activity.

Component analyses indicated that in the fully adjusted model, each 1-point increase in the score for gut microbiota–beneficial dietary components was associated with a 12% reduction in HF risk (OR = 0.88, 95% CI: 0.83–0.94, *P* < .001), whereas unfavourable components were not significantly associated with HF risk (*P* = .678). Further subgroup analyses (Supplementary Table S1) suggested that this association was mainly present in the prediabetes group: each 1-point increase in DI-GM was associated with an approximately 11% lower risk of HF (OR = 0.89, 95% CI: 0.82–0.96, *P* = .004), with a significant trend (*P* for trend = .009). No statistically significant association was observed in the diabetes group (OR = 0.97, 95% CI: 0.91–1.03, *P* = .333).

**Figure 2:**
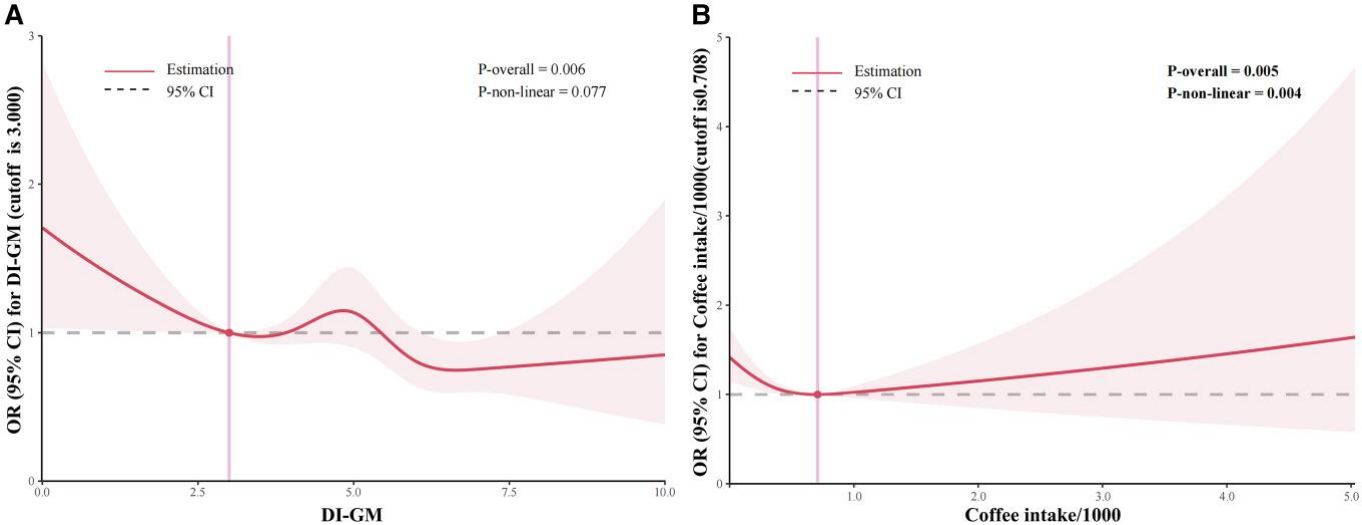
Associations of DI-GM and Coffee Intake with Heart Failure Risk. (*A*) Dose–response relationship between DI-GM and the risk of heart failure (*P*-overall = .006). (*B*) Dose–response relationship between coffee intake (per 1000 units) and the risk of heart failure (*P*-overall = .005, *P*-non-linear = .004). OR, odds ratio; CI, confidence interval

*Figure 2* further illustrated the overall pattern of the association. Results showed a significant linear relationship between DI-GM scores and HF risk (*P*-overall = .006), with no evidence of nonlinearity (*P*-nonlinear = .077). The lowest risk was observed at a DI-GM score of approximately 3.0, followed by a slight increase thereafter. Moreover, coffee intake showed a significant nonlinear association with HF risk (*P*-overall = .005, *P*-nonlinear = .004), with threshold analysis showing the lowest risk at an intake level of approximately 0.578 (unit: intake/1000), after which risk increased with higher intake (*Table 3*). Subgroup analyses (Supplementary Figure S1) indicated no significant association between DI-GM scores and HF risk among participants with diabetes (*P*-overall = .386), whereas an inverse association was observed in the prediabetes group (*P*-overall = .012), though without evidence of nonlinearity (*P*-nonlinear = .236). Collectively, these findings suggest that higher DI-GM scores may confer clearer protective effects at the prediabetes stage.

**Table 3:** Segmented logistic regression models were used to analyse the threshold effect of coffee intake/1000 on the risk of heart failure in adults with diabetes or prediabetes in NHANES from 2007 to 2018

Threshold effect analysis	OR (95% CI)	*P*-value
Model 1: Standard logistic regression model	0.839 (0.675–1.025)	.100
Model 2: Segmented logistic regression model		
Inflection point (0.578)		
Coffee intake/1000 ≤ 0.578	0.503 (0.336–0.753)	.**001**
Coffee intake/1000 > 0.578	1.148 (0.873–1.427)	.261
Log-likelihood ratio test		.**004**

Model adjusted for gender, age, race, education level, marital status, PIR, BMI, waist circumference, smoking status, alcohol consumption, TC, diabetes, hypertension, CHD, physical activity.

PIR, poverty income ratio; BMI, body mass index; TC, total cholesterol; CHD, coronary heart disease.

The distribution of glucose-lowering medication use across DI-GM quartiles was analysed (Supplementary Figure S2A), and further adjustment for medication use did not materially change the association between DI-GM and HF risk (Supplementary Figure S2B).

### Subgroup analyses

**Figure 3:**
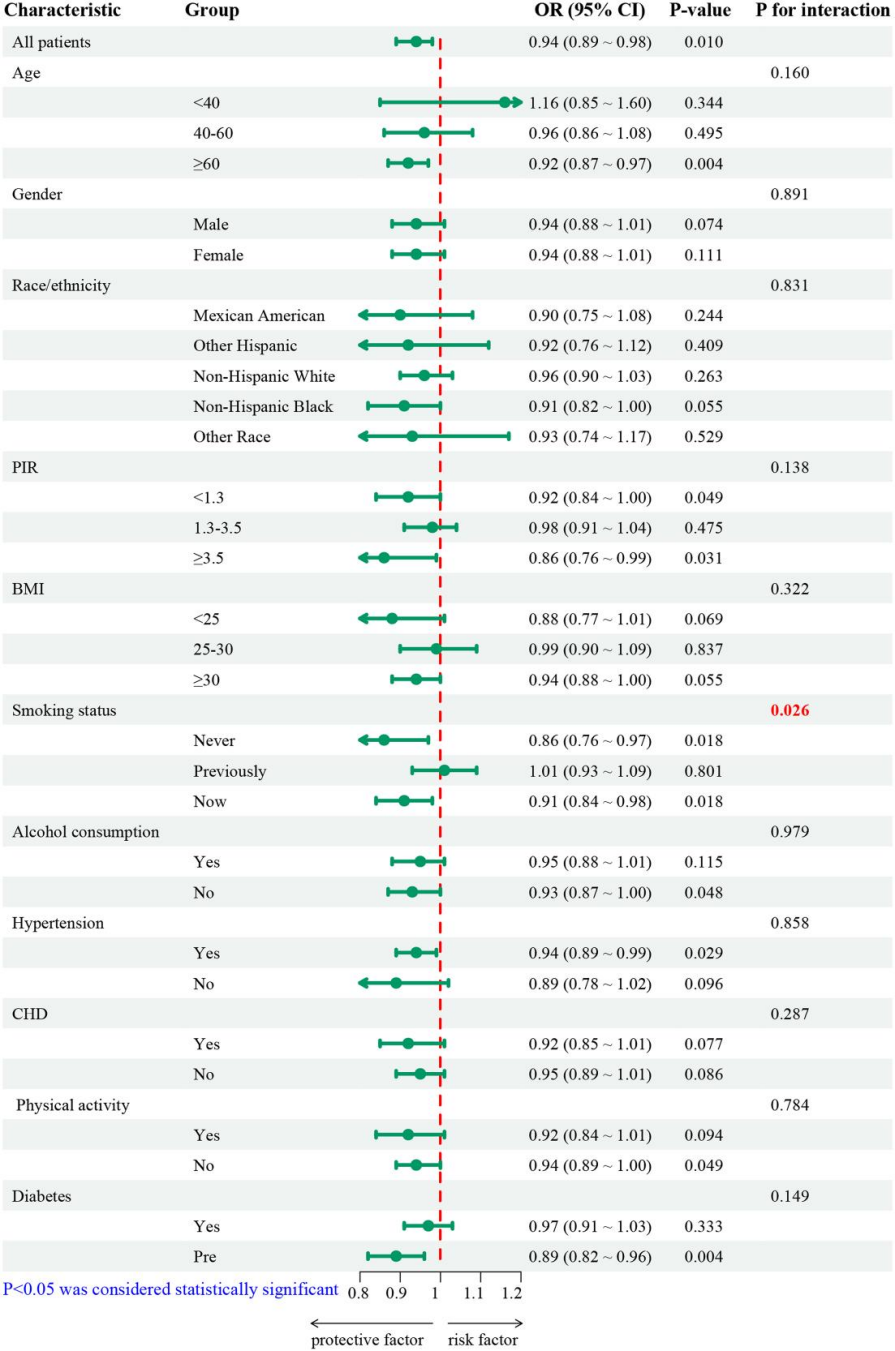
Subgroup analysis of the association between DI-GM and heart failure risk. Legend: This forest plot presents the association between DI-GM and the risk of heart failure across various population subgroups. The model was adjusted for potential confounding factors. The odds ratios (ORs) with 95% confidence intervals are shown for each subgroup. *P*-values were used to test for effect heterogeneity across subgroups. An OR < 1 indicates that DI-GM is a protective factor against heart failure. CHD, coronary heart disease; PIR, poverty-income ratio

In the overall population, higher DI-GM scores were significantly associated with a reduced risk of HF (OR = 0.94, 95% CI: 0.89–0.98, *P* = .010), and the direction of association was consistent across most subgroups, with no significant interactions (*P* for interaction > .05), suggesting a broadly applicable relationship (*Figure 3*). The inverse association appeared more pronounced in certain subgroups, such as participants aged ≥60 years (OR = 0.92, 95% CI: 0.87–0.97), those with PIR ≥3.5 (OR = 0.86, 95% CI: 0.76–0.99), and non-smokers (OR = 0.86, 95% CI: 0.76–0.97). Notably, smoking status showed a significant interaction (*P* = .026), indicating a potential modifying effect.

In stratified analyses by glycaemic status (Supplementary Figure S3), the association was particularly evident among participants with prediabetes: higher DI-GM scores were significantly associated with a lower prevalence of HF (OR = 0.89, 95% CI: 0.82–0.96, *P* = .004). Consistent inverse associations were observed across multiple subgroups, including women (OR = 0.88, 95% CI: 0.78–0.99), current smokers (OR = 0.81, 95% CI: 0.68–0.97), non-smokers (OR = 0.71, 95% CI: 0.61–0.82), and individuals without coronary heart disease (OR = 0.75, 95% CI: 0.62–0.90). Significant statistical interactions were identified for smoking status (*P* = .013) and coronary heart disease (*P* = .046).

By contrast, in the diabetes subgroup, the association between DI-GM scores and HF risk did not reach statistical significance (OR = 0.97, 95% CI: 0.91–1.03, *P* = .333).

Taken together, these findings indicate a generally inverse association between DI-GM scores and HF risk in the overall population, with a stronger effect observed among individuals with prediabetes, while no significant association was detected among those with established diabetes.

### LASSO regression analysis

**Figure 4:**
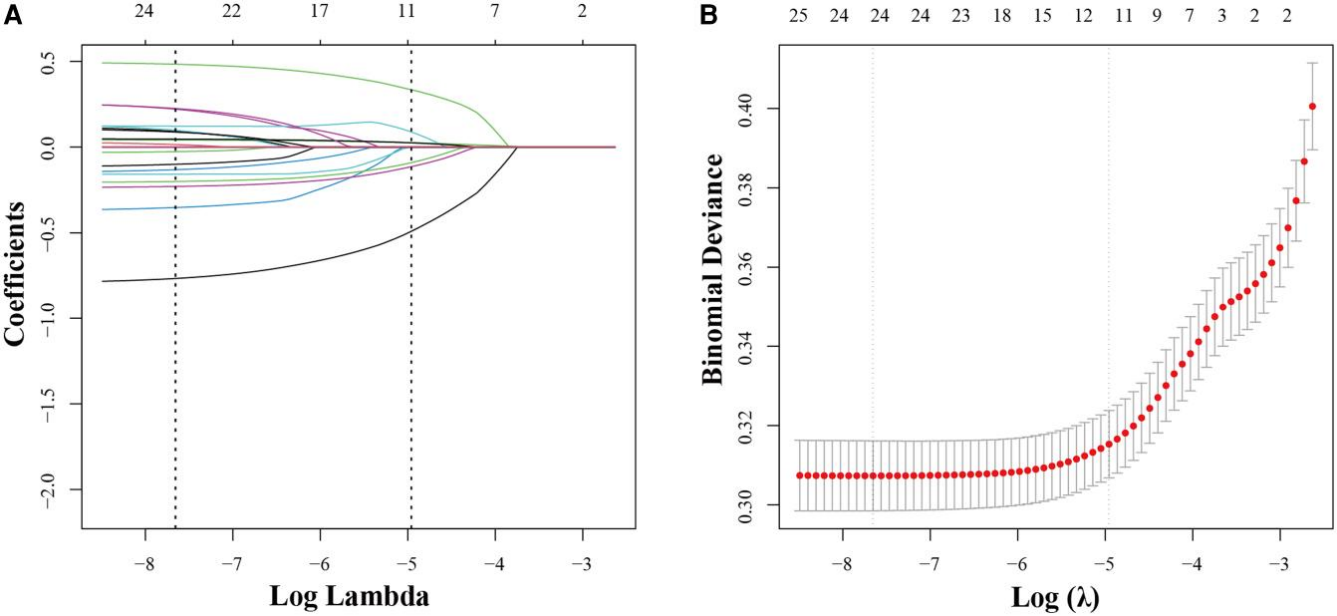
Feature selection for DI-GM using LASSO regression. (*A*) Coefficient Paths: Showing the shrinkage of variable coefficients towards zero as the regularization parameter (λ) increases. (*B*) Cross-Validation: Identifying the optimal lambda value (dashed line) based on the minimum error criterion

LASSO regression was applied to identify variables most relevant to HF (*Figure 4*). As shown in *Figure 4A*, increasing the penalty parameter λ gradually shrank some coefficients to zero, indicating limited contribution of those variables. The optimal λ was determined by 10-fold cross-validation (*Figure 4B*), and the parsimonious model at λ.1se retained only the most influential predictors.

The regression coefficients of the selected variables are summarized in Supplementary Table S2. Hypertension (r = 5.9891) and coronary heart disease (r = 7.8964) had the largest positive coefficients, while diabetes (r = −2.0288) and poverty income ratio (r = −0.1029) showed negative associations. Age (r = 0.0244) and BMI (r = 0.0230) were also retained with small positive effects. These results indicate that cardiovascular comorbidities and basic metabolic indicators are the main contributors in the final model.

### Model performance evaluation

**Figure 5:**
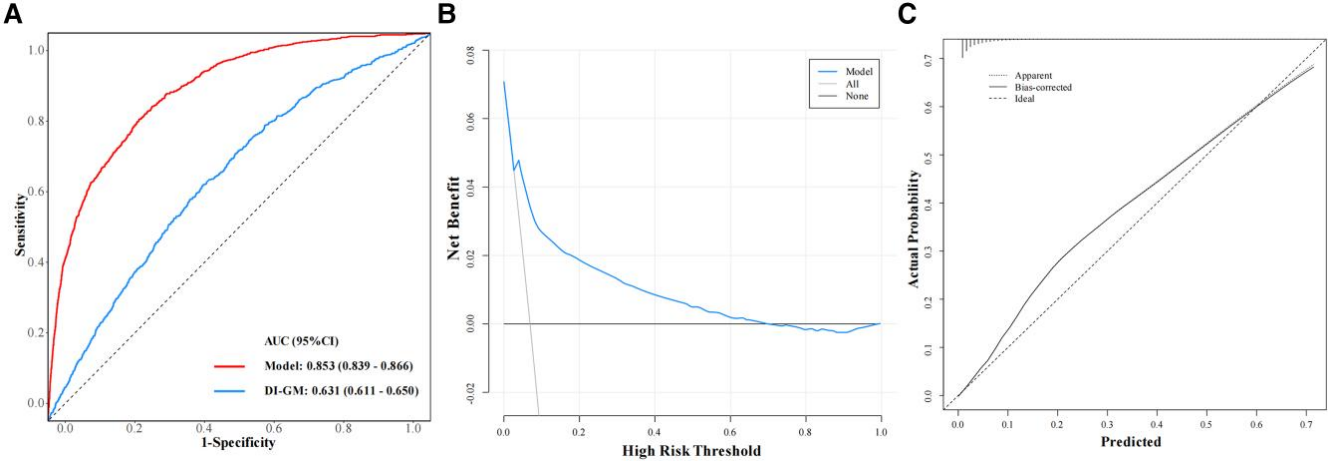
Performance evaluation of the heart failure risk prediction model. (*A*) ROC Curves: Comparing the predictive discrimination of the new model (incorporating DI-GM) against the baseline model. (*B*) Decision Curve: Assessing the clinical net benefit of the model across various decision thresholds. (*C*) Calibration Plot: Demonstrating the agreement between predicted probabilities and observed outcomes

The predictive value of DI-GM for HF was examined using a logistic regression model with multiple validation analyses (*Figs 5A–C*). The ROC results showed that the combined model had an AUC of 0.853 (95% CI: 0.839–0.866), clearly higher than that of DI-GM alone (AUC = 0.631, 95% CI: 0.611–0.650), indicating better overall prediction. Supplementary Table S3 summarizes the diagnostic performance of both models. Compared with DI-GM alone, the combined model achieved higher accuracy (0.763 vs. 0.597), sensitivity (0.763 vs. 0.596), and specificity (0.773 vs. 0.606). The positive predictive value also increased (0.984 vs. 0.966), with similar cutoff points (0.05 vs. 0.049). These findings suggest that incorporating demographic and clinical factors such as age, PIR, BMI, hypertension, diabetes, and CHD enhances the model's predictive performance for HF. As shown in *Figs 5B* and *5C*, the combined model yielded better decision outcomes across most probability ranges, and the predicted probabilities were close to the observed values, supporting consistent model performance.

## Discussion

This study, based on NHANES data from 2007 to 2018, provides robust evidence regarding the association between DI-GM and the risk of HF among individuals with diabetes and prediabetes. The findings indicate that a higher DI-GM score, which reflects a dietary pattern rich in dietary fibre, whole grains, and fermented foods, is significantly associated with a reduced risk of HF, even after comprehensive adjustment for multiple potential confounders. Notably, participants with a DI-GM score of ≥6 exhibited a markedly lower risk of HF. Restricted cubic spline analysis further demonstrated a gradual decline in HF risk with increasing DI-GM scores, with the protective effect being particularly prominent in individuals with prediabetes. In addition, coffee consumption showed a nonlinear relationship with HF risk, with moderate intake associated with the lowest risk. Sensitivity and subgroup analyses yielded highly consistent results, underscoring the reliability and reproducibility of the study findings.

### Comparison with previous studies

This study further advances the exploration of the role of gut microbiota in the risk of HF among individuals with diabetes and prediabetes. In a large-scale population-based analysis, higher DI-GM scores were significantly associated with a lower risk of HF among individuals with dysglycaemia. This finding provides important population-based evidence for the ‘gut–heart axis’ hypothesis.^11,12^ Unlike previous studies that primarily focused on single metabolites such as TMAO or specific bacterial taxa,^11^ our study emphasizes the cumulative effects of overall dietary patterns on the gut microbiota, which may operate through multiple pathways, including improved microbial function and reduced systemic inflammation.^12,13^ Focusing on the interplay between the gastrointestinal system and HF, the ‘gut–heart axis’ concept emphasizes that HF can induce intestinal hypoperfusion and congestion, thereby impairing gut barrier integrity and triggering inflammatory responses. Meanwhile, gut microbiota–derived metabolites (such as trimethylamine N-oxide, TMAO) can enter the circulation and contribute to cardiovascular injury.^14^ As a key product of choline and carnitine metabolism, TMAO has been associated with disease severity and adverse outcomes across various acute and chronic HF populations.^14,15^ Notably, this association was most pronounced in individuals with prediabetes, suggesting that dietary interventions may be particularly effective for preventing HF at the early stages of disease. The results suggest a certain degree of heterogeneity across different population subgroups. Previous studies have shown that the prognostic value of TMAO varies by ethnicity, with a more pronounced effect observed in individuals of European ancestry.^16^

Both mechanistic and epidemiological evidence support our findings: diets rich in fermentable dietary fibre promote short-chain fatty acid production, which improves inflammation and gut barrier function, and influences blood pressure and cardiac remodeling^17–19^; fermented foods help enhance microbial diversity and metabolic capacity^20–22^; in contrast, high consumption of red meat and choline/carnitine may promote TMAO production, thereby increasing the risk of cardiovascular events.^23^ Multiple prior cohort studies and systematic reviews have also reported that intake of whole grains and dietary fibre is associated with reduced cardiovascular events and mortality^24^; fermented foods or probiotic-containing dairy products have been linked to improvements in metabolic profiles,^25^ whereas elevated TMAO levels are associated with adverse cardiovascular outcomes.^26^ Recently proposed gut–heart axis–related indices have attempted to integrate dietary structure and microbiota-derived metabolic effects into a unified metric.^27^ Compared with analyses focusing on individual nutrients, such approaches are better suited to capturing the relationship between overall dietary patterns and cardiovascular risk, thereby offering new perspectives for population stratification and intervention studies.

### Subgroup-specific effects

The subgroup analyses revealed heterogeneity in the inverse association between DI-GM scores and the risk of HF, suggesting that individual characteristics may modulate the effects of the ‘diet–microbiota–heart’ pathway. The consistent results observed in the overall population indicate that a dietary pattern characterized by high intake of dietary fibre and fermented foods may reduce the risk of HF by improving gut–heart interactions. Notably, smoking markedly attenuated the protective association between higher DI-GM scores and HF risk. This phenomenon may be explained by smoking-induced systemic inflammation, oxidative stress, and impaired gut barrier function, which could offset the beneficial effects of diet on gut microbiota and host metabolism.^28^

More importantly, our findings demonstrated that the inverse association between DI-GM scores and HF risk was strongest in individuals with prediabetes but substantially weaker among those with established diabetes. This observation suggests that dietary interventions may be most effective at the early stage of metabolic dysregulation, whereas the progression of diabetes and the accompanying gut dysbiosis and metabolic disturbances may diminish these benefits.^29^

In addition, the significant association observed among participants with higher socioeconomic status highlights the role of food accessibility, nutritional awareness, and other social–behavioural factors in shaping the effectiveness of dietary interventions.^30^ Taken together, our study suggests that future prevention strategies based on DI-GM should account for metabolic status, smoking history, and socioeconomic factors to develop more targeted and individualized nutritional strategies aimed at improving the effectiveness of diet-based interventions in the primary prevention of cardiovascular diseases.

### Mechanistic insights

To further interpret these subgroup-specific effects, potential mechanistic pathways should be considered. The gut microbiota may influence the risk of HF in individuals with diabetes and prediabetes through multiple pathways. Our study further revealed that the inverse association between DI-GM and HF risk was most evident in the prediabetic population, whereas this association was markedly attenuated among patients with established diabetes, suggesting that dietary interventions may have an optimal preventive window for HF. At the prediabetic stage, the DI-GM dietary pattern may exert cardiovascular protective effects through several mechanisms: First, by promoting the growth and metabolic activity of short-chain fatty acid (SCFA)-producing bacteria and increasing beneficial metabolites such as acetate and butyrate, thereby improving insulin sensitivity and glucose homeostasis.^31^ Second, by enhancing gut barrier integrity and reducing chronic low-grade inflammation induced by gut-derived endotoxins, thereby mitigating their detrimental effects on cardiac function.^32^ Third, by modulating the bile acid profile and activating related receptor signalling pathways (e.g. FXR and TGR5), which help maintain energy balance and suppress inflammatory responses.^33^ Thus, dietary modulation of the gut microbiota may confer more substantial cardiovascular benefits at the prediabetic stage, whereas its potential benefits may become limited once diabetes is established.

### Strengths, limitations, and future directions

One of the major strengths of this study lies in the use of NHANES, a nationally representative large-scale database, through which we, for the first time, clearly demonstrated that the relationship between DI-GM and HF risk varies significantly across different states of glucose metabolism—being particularly pronounced in individuals with prediabetes, while markedly attenuated among those with established diabetes. This finding not only provides robust population-based evidence for the ‘gut–heart axis’ hypothesis but also highlights the critical importance of timing in dietary interventions for HF prevention. In our analyses, we rigorously controlled for a wide range of sociodemographic, behavioural, and metabolic confounders, and further confirmed the consistency and reliability of the results through multiple subgroup and sensitivity analyses, thereby strengthening the persuasiveness and clinical relevance of our conclusions.

Of course, this study also has several limitations. First, given the cross-sectional design, we cannot establish a causal relationship between DI-GM and HF, and residual confounding factors may still have influenced the results. Second, the DI-GM score was derived from two 24-hour dietary recalls. Although this approach may reduce within-person variability to some extent, it remains subject to recall bias and may not adequately capture long-term dietary patterns. More importantly, the NHANES database does not provide measurements of gut microbiota composition or related circulating metabolites (such as short-chain fatty acids and TMAO), which limits our ability to directly validate the hypothesized mechanistic pathways linking diet, microbiota, and HF. After further adjustment for glucose-lowering medication use, the results remained largely unchanged, suggesting that potential confounding by treatment may be limited. In addition, since this study was conducted in a U.S. population, the generalizability of the findings to populations with different dietary cultures or ethnic backgrounds requires further investigation.

Despite these limitations, the findings of this study may have potential implications for clinical practice. As an index reflecting overall dietary patterns, DI-GM may serve as a useful tool for assisting in the assessment of HF risk among individuals with diabetes and prediabetes. In practice, increasing the intake of dietary fibre, whole grains, and fermented foods, while reducing the consumption of red meat and processed foods, may help improve the metabolic milieu and thereby potentially lower the risk of HF. However, its clinical utility warrants further validation in future studies.

## Conclusion

This study, for the first time, demonstrated using large-scale population data that the DI-GM–related dietary pattern (characterized by high intake of dietary fibre, whole grains, and fermented foods) confers the strongest protective effect against HF during the prediabetic stage, whereas this effect is markedly attenuated after diabetes is established, underscoring the critical importance of early nutritional intervention. Leveraging a nationally representative large sample, we verified a consistent and reliable dose–response association through multivariable adjustments and sensitivity analyses, thereby overcoming the limitations of prior studies that focused solely on individual nutrients or specific microbial taxa, and providing novel insights and practical strategies for HF prevention via dietary modulation of the gut microbiota.

## Supplementary Material

xvag125_Supplementary_Data

## Declarations

### Data Availability

The datasets analysed during the current study are publicly available from the NHANES website: https://www.cdc.gov/nchs/nhanes/.

### Pre-registered Clinical Trial Number

None supplied.
