# Supplementary material for: Diet–gut microbiota interaction Index and heart failure risk in diabetes and prediabetes: evidence from NHANES 2007–2018
Source: ESC Heart Fail. 2026 May 4;13(3):xvag125. doi: 10.1093/eschf/xvag125 (PMC13247595; doi:10.1093/eschf/xvag125)
Supplement: xvag125_Supplementary_Data [file xvag125_supplementary_data.zip › Supplementary_Material_Revised_Clean.docx]

Supplementary Figures and Tables

**Table S1** Relationship between DI-GM and odds of heart failure in diabetic or prediabetic participants.

| **Characteristic** | | | **Model** **1^a^** | | **Model 2** **^b^** | | **Model 3 ^c^** | |
| --- | --- | --- | --- | --- | --- | --- | --- | --- |
|  |  |  | **OR (95% CI)** | **P-value** | **OR (95% CI)** | **P-value** | **OR (95% CI)** | **P-value** |
| **Diabetes participants** | | | | | | | | |
| **Continuous DI-GM** | | | 0.95 (0.89, 1.01) | 0.074 | 0.94 (0.88, 0.99) | **0.030** | 0.97 (0.91, 1.03) | 0.333 |
| **DI-GM** | 1 (0-3 score) | | Ref |  | Ref |  | Ref |  |
|  | 2 (4 score) | | 0.84 (0.65, 1.09) | 0.192 | 0.86 (0.66, 1.12) | 0.261 | 0.92 (0.69, 1.23) | 0.592 |
|  | 3 (5 score) | | 1.06 (0.82, 1.37) | 0.645 | 1.05 (0.80, 1.36) | 0.733 | 1.11 (0.83, 1.47) | 0.489 |
|  | 4 (≥6 score) | | 0.79 (0.61, 1.03) | 0.078 | 0.75 (0.57, 0.97) | **0.032** | 0.86 (0.64, 1.15) | 0.298 |
|  | P for trend | | 0.95 (0.88, 1.03) | 0.247 | 0.93 (0.86, 1.01) | 0.107 | 0.97 (0.89, 1.07) | 0.545 |
| Beneficial to gut microbiota | | | 0.89 (0.83, 0.96) | **0.002** | 0.90 (0.83, 0.97) | **0.004** | 0.93 (0.85, 1.01) | 0.068 |
| Unfavorable to gut microbiota | | | 1.05 (0.96, 1.14) | 0.284 | 1.01 (0.93, 1.10) | 0.810 | 1.03 (0.94, 1.14) | 0.481 |
| **Prediabetes participants** | | | | | | | | |
| **Continuous DI-GM** | | | 0.91 (0.85, 0.98) | **0.014** | 0.87 (0.80, 0.93) | **<0.001** | 0.89 (0.82, 0.96) | **0.004** |
| **DI-GM** | | 1 (0-3 score) | Ref |  | Ref |  | Ref |  |
|  |  | 2 (4 score) | 1.13 (0.82, 1.55) | 0.464 | 1.11 (0.80, 1.53) | 0.538 | 1.11 (0.79, 1.56) | 0.557 |
|  |  | 3 (5 score) | 1.05 (0.75, 1.45) | 0.792 | 0.98 (0.70, 1.36) | 0.891 | 1.02 (0.71, 1.45) | 0.929 |
|  |  | 4 (≥6 score) | 0.70 (0.49, 0.98) | **0.038** | 0.54 (0.38, 0.77) | **<0.001** | 0.62 (0.43, 0.90) | **0.011** |
|  |  | P for trend | 0.89 (0.81, 0.99) | **0.030** | 0.83 (0.74, 0.92) | **<0.001** | 0.86 (0.77, 0.96) | **0.009** |
| Beneficial to gut microbiota | | | 0.83 (0.75, 0.91) | **<0.001** | 0.80 (0.73, 0.88) | **<0.001** | 0.82 (0.74, 0.91) | **<0.001** |
| Unfavorable to gut microbiota | | | 1.08 (0.97, 1.21) | 0.177 | 0.98 (0.87, 1.10) | 0.714 | 1.02 (0.90, 1.14) | 0.795 |

OR: odds ratio.

95% CI: 95% confidence interval.

^a^ Model 1: no covariates were adjusted.

^b^ Model 2: adjusted for gender, age, and race.

^c^ Model 3: adjusted for gender, age, race, education level, marital status, PIR, BMI, waist circumference, smoking status, alcohol consumption, TC, diabetes, hypertension, CHD, physical activity.

Abbreviations: DI-GM, dietary index for gut microbiota; PIR, poverty income ratio; BMI: body mass index; TC, total cholesterol; CHD: coronary heart disease.

**Table S2** Lasso regression analysis results (best log(λ) = -4.9546).

| **Characteristic** | **r** | **Characteristic** | **r** |
| --- | --- | --- | --- |
| Gender | / | PIR | -0.1029 |
| Race | / | Education level | / |
| Marital status | / | BMI | 0.0230 |
| TC | / | Smoking status | / |
| Waist circumference | / | Hypertension | 5.9891 |
| Physical activity | / | Diabetes | -2.0288 |
| Alcohol consumption | / | CHD | 7.8964 |
| Age | 0.0244 |  |  |

Abbreviations: PIR, poverty income ratio; BMI: body mass index; TC, total cholesterol; CHD: coronary heart disease.

**Table S3** ROC was used to analyze the diagnostic efficacy of DI-GM and the model for heart failure in diabetes and prediabetes. The model was based on DI-GM and adjusted for age, PIR, BMI, hypertension, diabetes, and CHD.

|  | AUC (95%CI) | Accuracy (95%CI) | Sensitivity (95%CI) | Specificity (95%CI) | PPV (95%CI) | NPV (95%CI) | Cut off |
| --- | --- | --- | --- | --- | --- | --- | --- |
| DI-GM | 0.631 (0.611-0.650) | 0.597 (0.589-0.605) | 0.596 (0.588 - 0.604) | 0.606 (0.572 - 0.641) | 0.966 (0.962 - 0.970) | 0.074 (0.068 - 0.081) | 0.049 |
| Model | 0.853 (0.839-0.866) | 0.763 (0.756-0.770) | 0.763 (0.756 - 0.770) | 0.773 (0.744 - 0.803) | 0.984 (0.982 - 0.987) | 0.148 (0.137 - 0.159) | 0.05 |

**Supplemental Figure Legends**

**Figure S1.** Dose-Response Relationships Between DI-GM and Heart Failure Risk, Stratified by Diabetes Status

**Figure S2.** Glucose-lowering medication across DI-GM quartiles and sensitivity analysis of its impact on the association between DI-GM and heart failure

(A) Distribution of insulin and oral hypoglycemic agents across DI-GM quartiles.

(B) Sensitivity analysis of the association between DI-GM and heart failure after adjustment for glucose-lowering medications.

**Figure S3.** Subgroup Analysis of the Association Between DI-GM and Heart Failure Risk, Stratified by Diabetes Status

**Figure S1.** Dose-Response Relationships Between DI-GM and Heart Failure Risk, Stratified by Diabetes Status


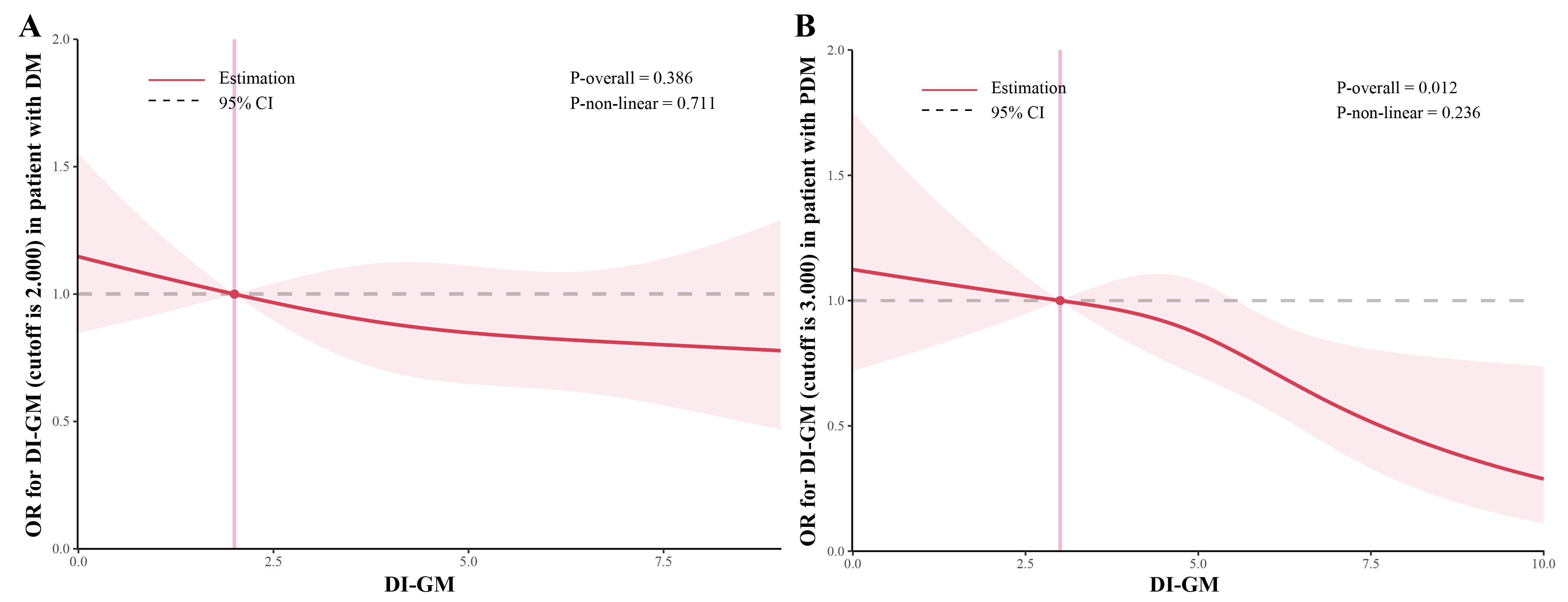


(A) Patients with diabetes: No significant non-linear or overall association was observed between DI-GM and heart failure risk.

(B) Patients with prediabetes: A significant overall association was observed between DI-GM and heart failure risk.

**Figure S2.** Glucose-lowering medication across DI-GM quartiles and sensitivity analysis of its impact on the association between DI-GM and heart failure

(A)
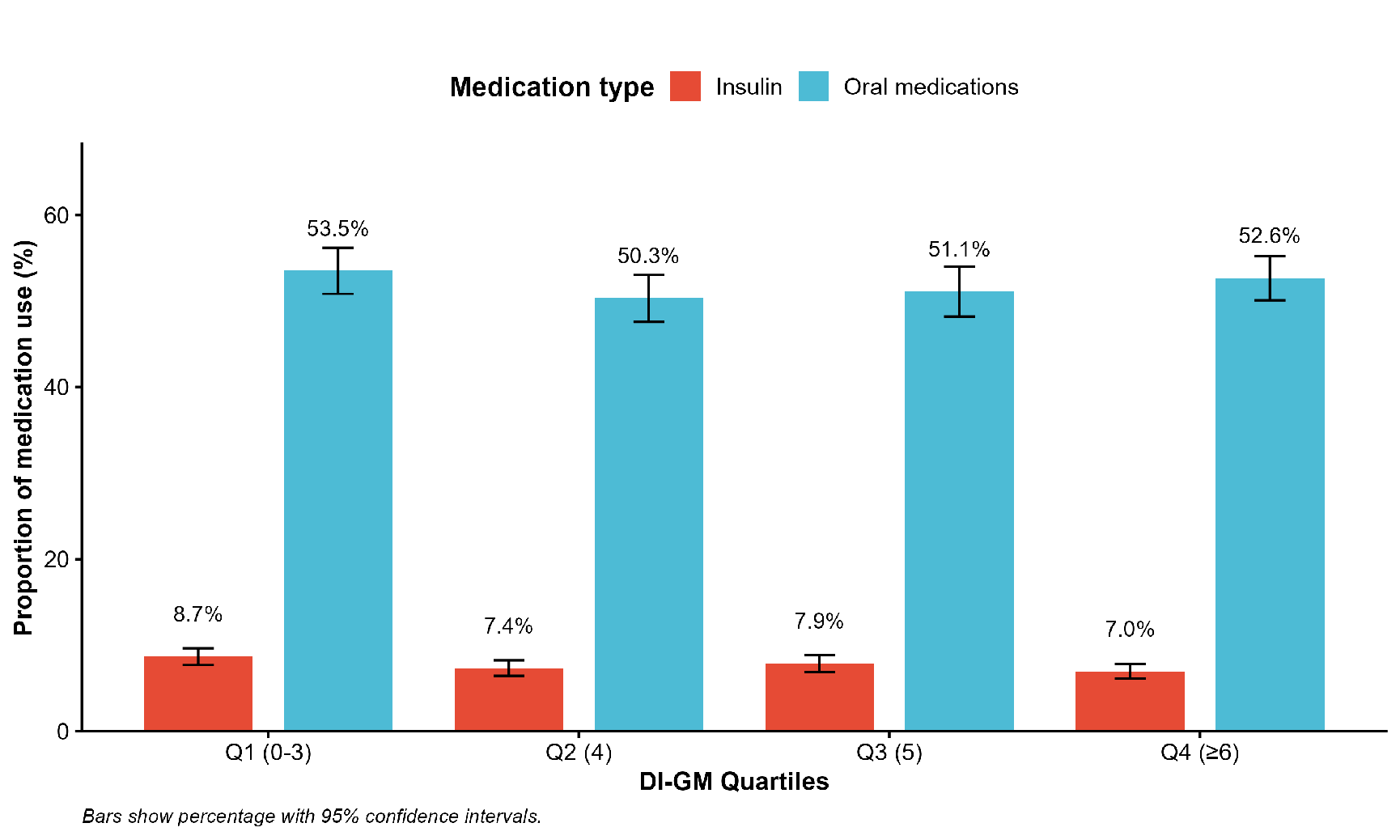


(B)
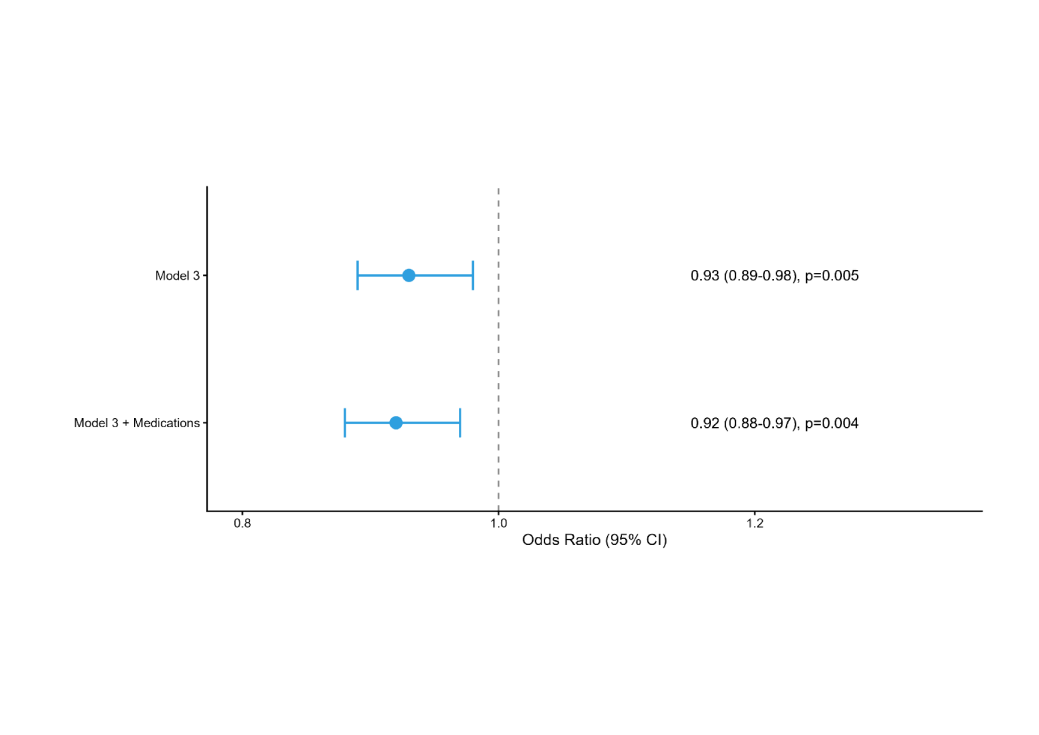


Legend:

(A) Distribution of insulin and oral hypoglycemic agents across DI-GM quartiles.

The bar charts represent the percentage of participants using insulin (red) and oral hypoglycemic agents (blue) within each DI-GM quartile. Error bars indicate 95% confidence intervals.

(B) Sensitivity analysis of the association between DI-GM and heart failure after adjustment for glucose-lowering medications.

Odds ratios (ORs) and 95% confidence intervals (CIs) are shown for the fully adjusted model (Model 3) and the model additionally adjusted for glucose-lowering medications (Model 3 + Medications), with similar estimates observed after adjustment.

**Figure S3.** Subgroup Analysis of the Association Between DI-GM and Heart Failure Risk, Stratified by Diabetes Status


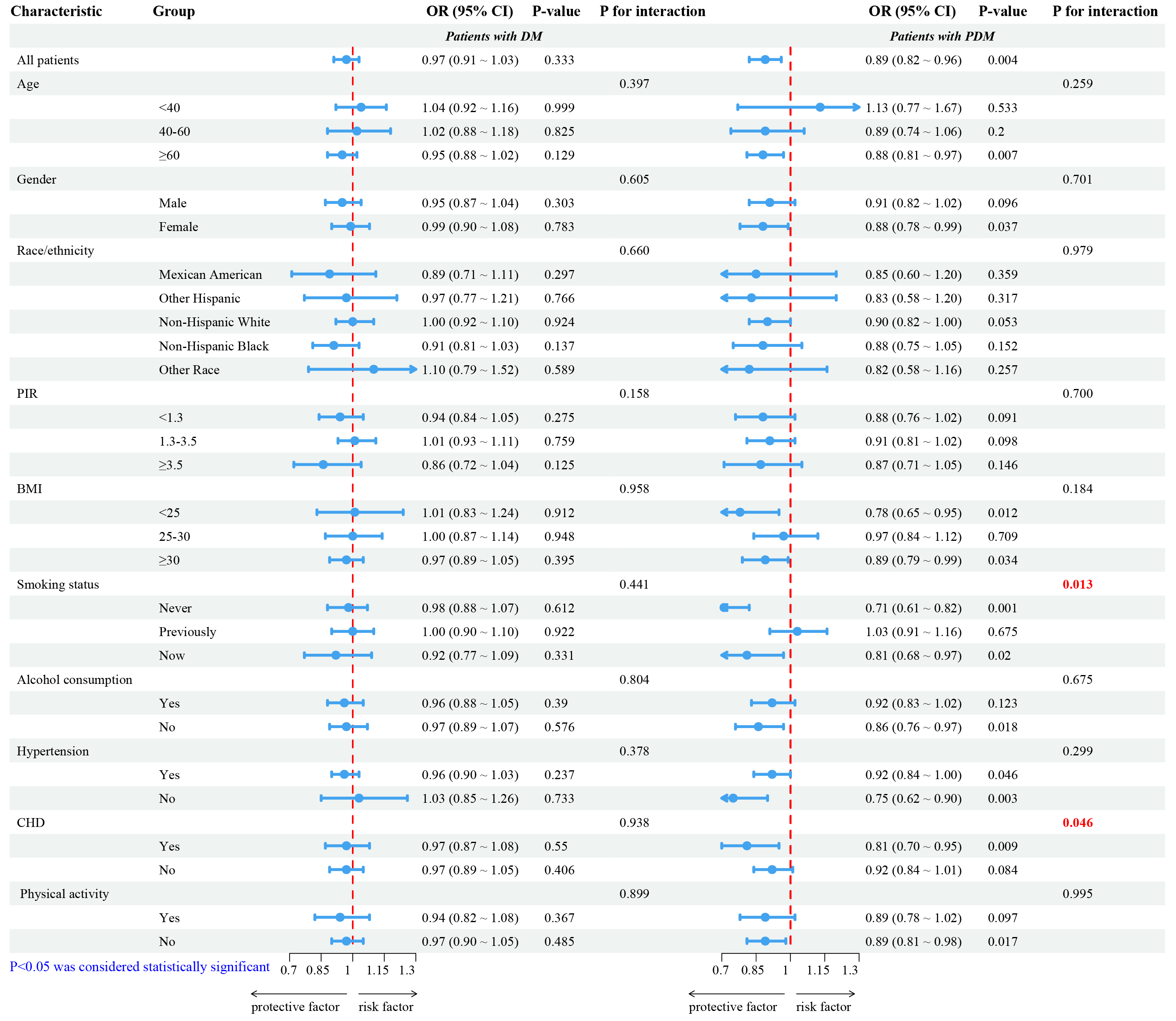


Legend: Forest plots present the association of DI-GM with heart failure risk across subgroups in participants with diabetes and those with prediabetes. All models were adjusted for multiple covariates. Odds ratios with 95% confidence intervals are denoted by squares and horizontal lines. An OR < 1 indicates a protective effect. P-values evaluate the interaction across subgroups. CHD, coronary heart disease; PIR, poverty-income ratio.
