# Supplementary figures and images for: Diet–gut microbiota interaction Index and heart failure risk in diabetes and prediabetes: evidence from NHANES 2007–2018
Source: ESC Heart Fail. 2026 May 4;13(3):xvag125. doi: 10.1093/eschf/xvag125 (PMC13247595; doi:10.1093/eschf/xvag125)

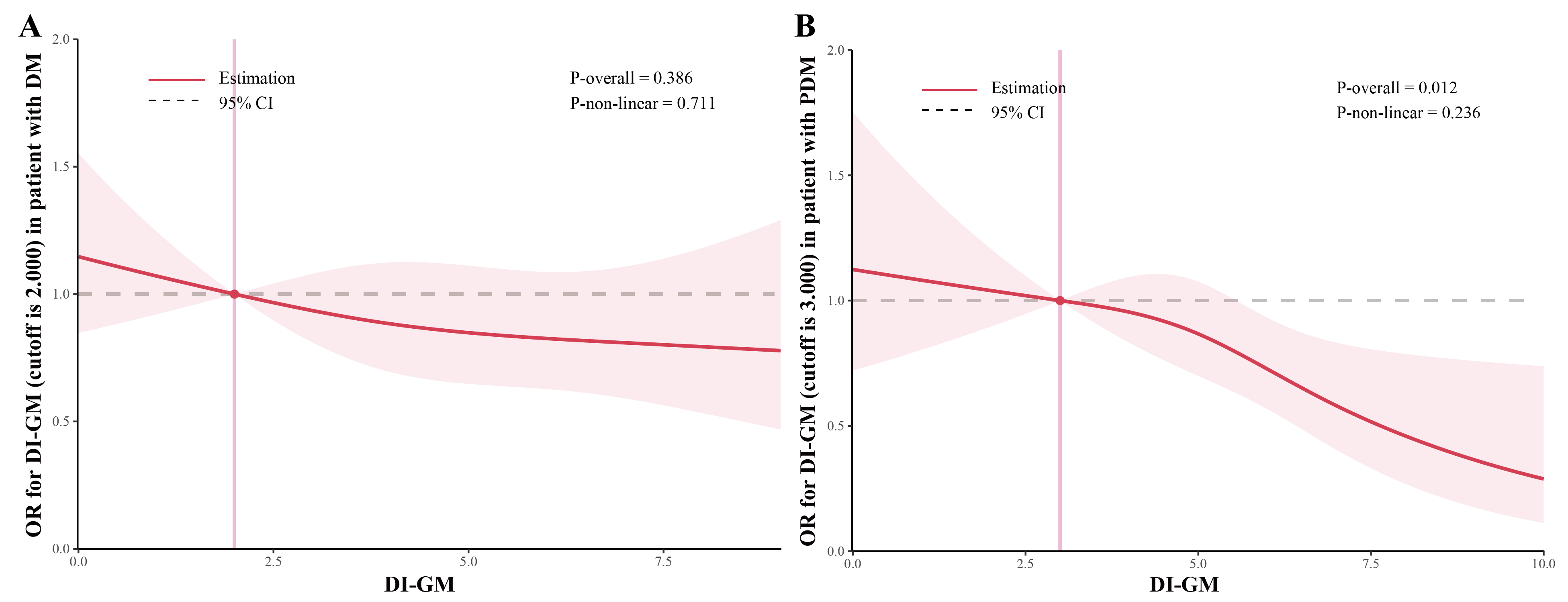

Supplement: xvag125_Supplementary_Data [file xvag125_supplementary_data.zip › Fig.S1.tif]

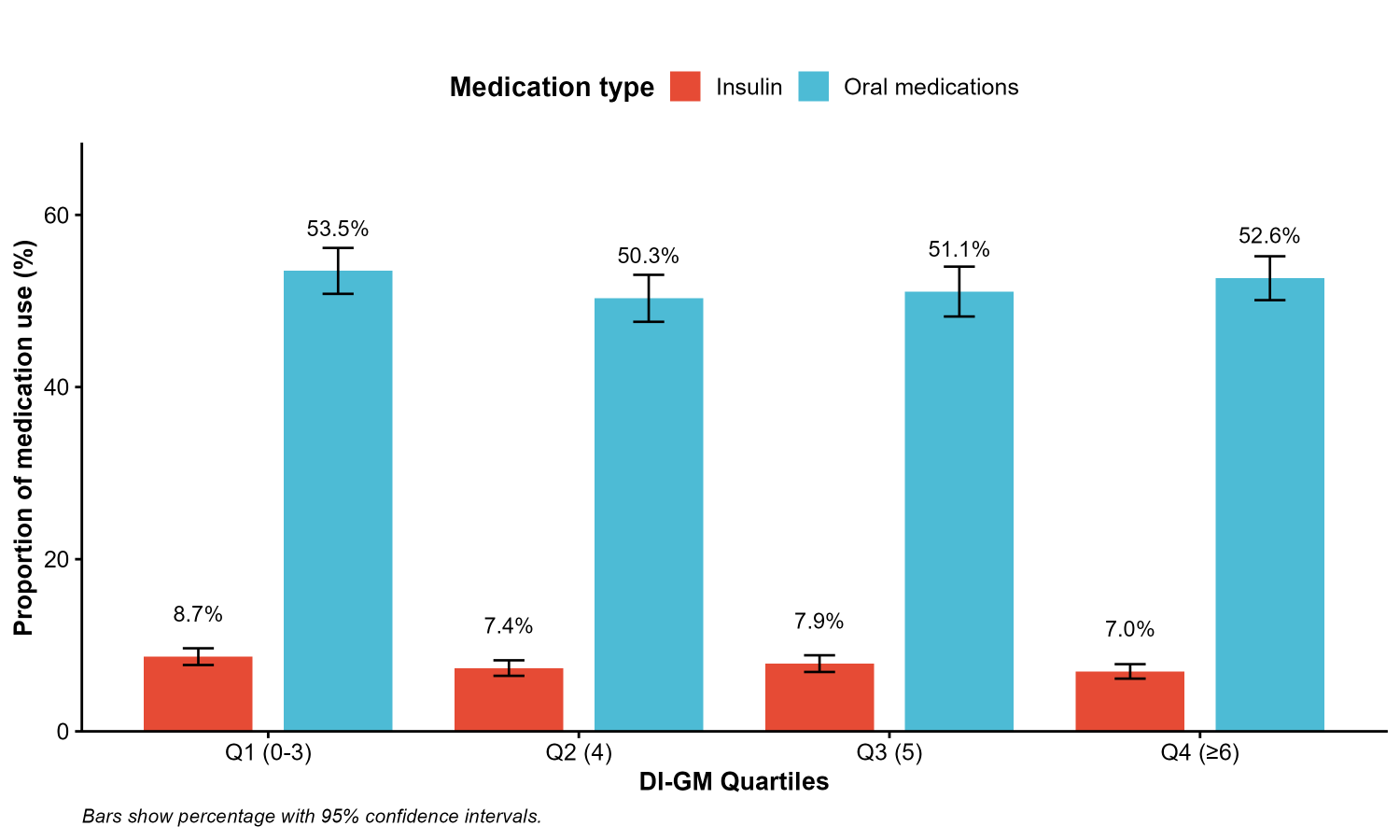

Supplement: xvag125_Supplementary_Data [file xvag125_supplementary_data.zip › Fig.S2A.tiff]

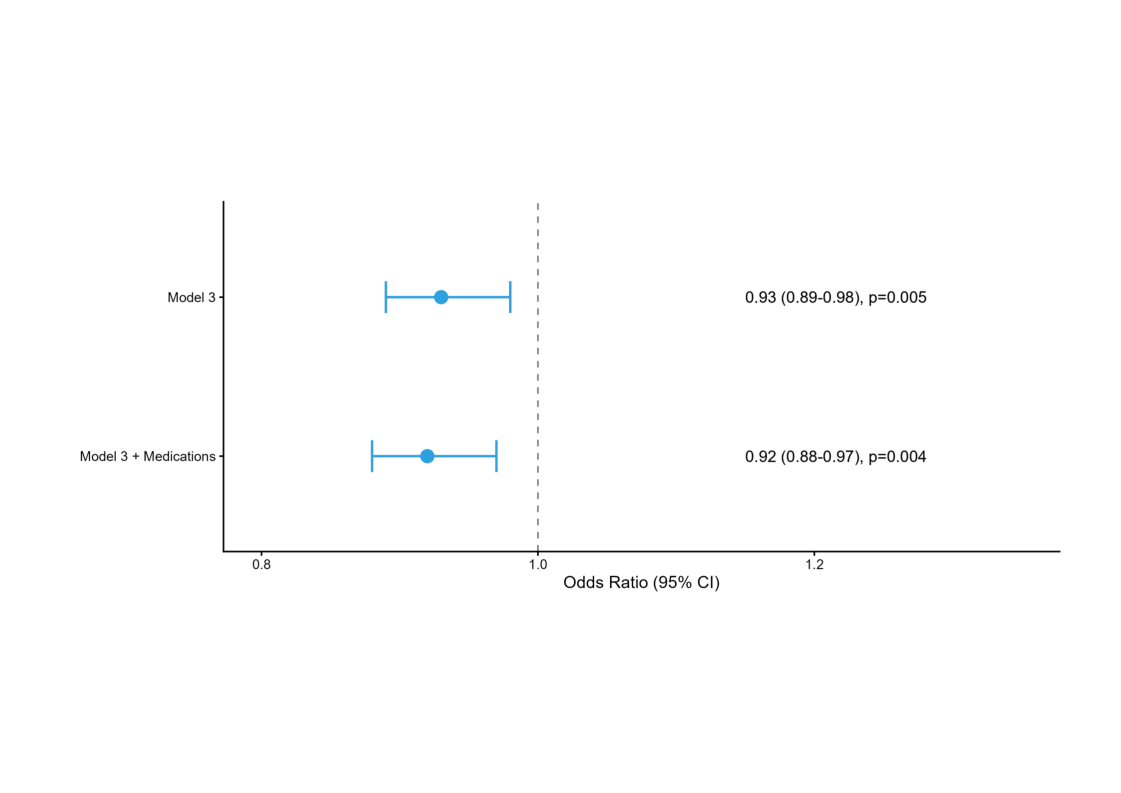

Supplement: xvag125_Supplementary_Data [file xvag125_supplementary_data.zip › Fig.S2B.tiff]

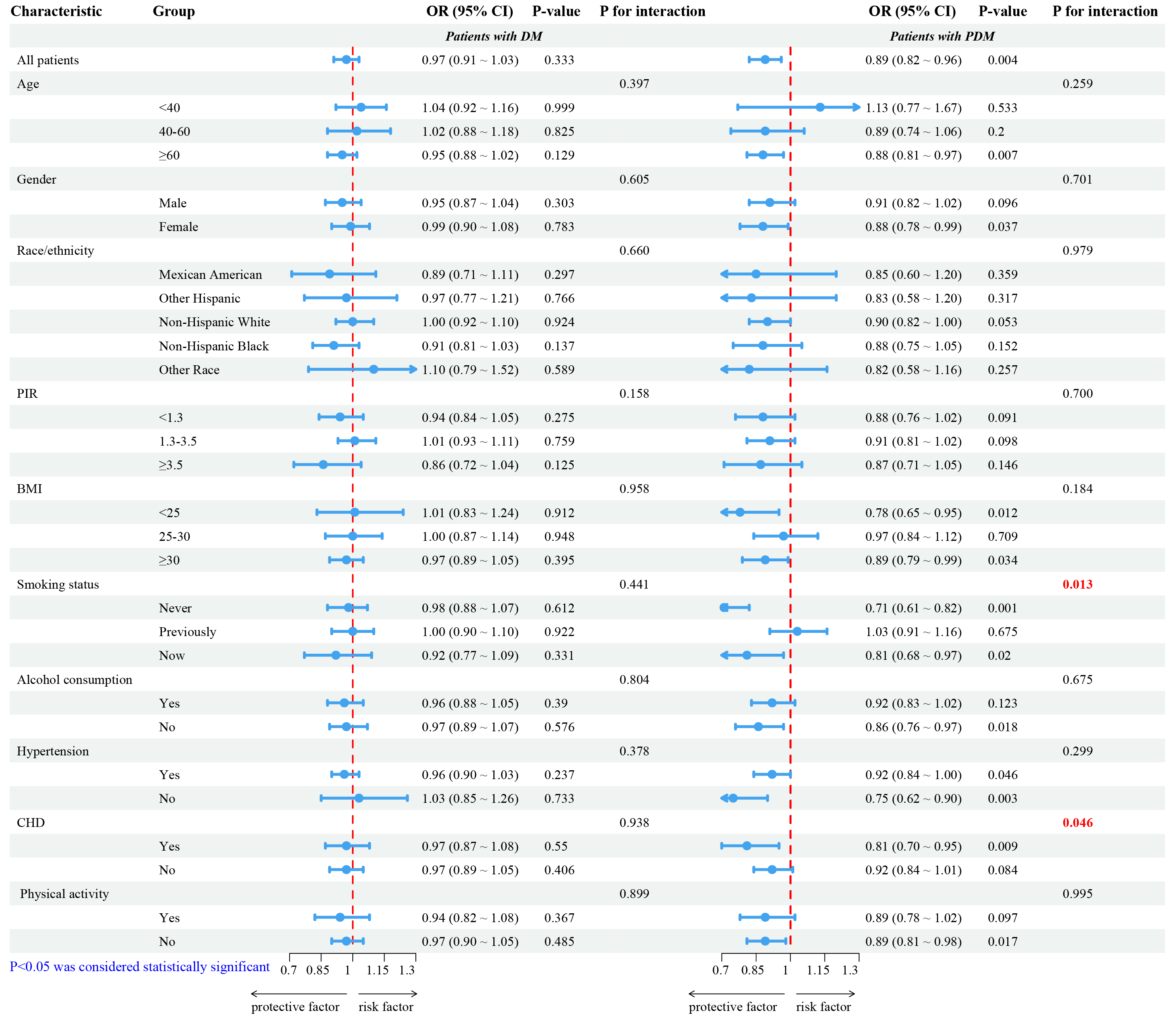

Supplement: xvag125_Supplementary_Data [file xvag125_supplementary_data.zip › Fig.S3.tif]
